# A new scoring system in Cystic Fibrosis: statistical tools for database analysis – a preliminary report

**DOI:** 10.1186/1472-6947-8-44

**Published:** 2008-10-05

**Authors:** GM Hafen, C Hurst, J Yearwood, J Smith, Z Dzalilov, PJ Robinson

**Affiliations:** 1 Department of Respiratory Medicine, Royal Children's Hospital Melbourne, Parkville, Victoria, Australia; 2 School of Information Technology and Mathematical Science, University of Ballarat, Ballarat, Victoria, Australia; 3 Department of Paediatrics, University of Melbourne, Melbourne, Victoria, Australia; 4 Centre for Informatics and Applied Optimization, University of Ballarat, Ballarat, Victoria, Australia; 5 Respiratory Unit, Department of Pediatrics, University Hospital CHUV, Lausanne, Switzerland

## Abstract

**Background:**

Cystic fibrosis is the most common fatal genetic disorder in the Caucasian population. Scoring systems for assessment of Cystic fibrosis disease severity have been used for almost 50 years, without being adapted to the milder phenotype of the disease in the 21^st ^century. The aim of this current project is to develop a new scoring system using a database and employing various statistical tools. This study protocol reports the development of the statistical tools in order to create such a scoring system.

**Methods:**

The evaluation is based on the Cystic Fibrosis database from the cohort at the Royal Children's Hospital in Melbourne. Initially, unsupervised clustering of the all data records was performed using a range of clustering algorithms. In particular incremental clustering algorithms were used. The clusters obtained were characterised using rules from decision trees and the results examined by clinicians. In order to obtain a clearer definition of classes expert opinion of each individual's clinical severity was sought. After data preparation including expert-opinion of an individual's clinical severity on a 3 point-scale (mild, moderate and severe disease), two multivariate techniques were used throughout the analysis to establish a method that would have a better success in feature selection and model derivation: 'Canonical Analysis of Principal Coordinates' and 'Linear Discriminant Analysis'. A 3-step procedure was performed with (1) selection of features, (2) extracting 5 severity classes out of a 3 severity class as defined per expert-opinion and (3) establishment of calibration datasets.

**Results:**

(1) Feature selection: CAP has a more effective "modelling" focus than DA.

(2) Extraction of 5 severity classes: after variables were identified as important in discriminating contiguous CF severity groups on the 3-point scale as mild/moderate and moderate/severe, Discriminant Function (DF) was used to determine the new groups mild, intermediate moderate, moderate, intermediate severe and severe disease. (3) Generated confusion tables showed a misclassification rate of 19.1% for males and 16.5% for females, with a majority of misallocations into adjacent severity classes particularly for males.

**Conclusion:**

Our preliminary data show that using CAP for detection of selection features and Linear DA to derive the actual model in a CF database might be helpful in developing a scoring system. However, there are several limitations, particularly more data entry points are needed to finalize a score and the statistical tools have further to be refined and validated, with re-running the statistical methods in the larger dataset.

## Background

Cystic fibrosis (CF) is the most common fatal genetic disorder in the Caucasian population, with a carrier rate of approximately five percent and an annual incidence of one in 2,500 live births [1]. This autosomal recessive disorder is caused by mutations in a single gene located on chromosome 7, which encodes for the cystic fibrosis transmembrane regulator protein (CFTR). More than 1500 different mutations have been identified [2]. Abnormalities in the CFTR leads to decreased chloride secretion and increased sodium absorption in these cells, resulting in dehydration of the airways surface layer and subsequent viscous tenacious mucus.

CF is a multi-system disease with a wide variability in the severity of symptoms and consequently the progression of the disease. Primarily it is the lungs and pancreas that are affected, with the small airways of the lungs and the pancreatic ducts being obstructed with the viscous tenacious mucus. This then often results in chronic lung disease and insufficient function of the exocrine pancreas. Despite the life expectancy increasing, where the median survival rates are now exceeding 30 years of age, [1] CF remains an incurable illness with over 90 percent of the deaths attributed to lung failure and its associated complications.

Scoring systems for assessment of CF illness severity have been used for almost 50 years, with the first clinical score published by Shwachman and Kulczycki in 1958 (SK-score) [3]. A numerical classification of CF individuals according to their score launched a more uniform assessment and contributed essentially towards better care and success of clinical trials [4]. A recent detailed review of scoring systems (exclusive radiological scores) from our group highlighted problems associated with the current systems available, particularly with the most widely used SK-score [5]. But despite controversial opinions regarding the use of scoring systems, there seems to be an ongoing demand for a system to assess the phenotype of this heterogenic disease for inter-individual comparison and intra-individual longitudinal follow up [5]. In particular, a validated system as a uniform outcome measure allow meaningful comparison between studies, hence giving the physician the possibility to classify the patients accordingly to a score. The underlying goal of our present research is to develop such a new scoring system, taking into account items regularly and repetitive assessed at the patient's visit inclusive non-respiratory issues such as nutrition and bacterial colonisation, and reflecting the broad spectrum from mild to severe disease status of children and adolescents with CF. The advantage of an validated scoring system over purely radiological scoring system as those using High Resolution Computer Tomography (HRCT) [6] or even hyperpolarized gas MRI [7] is that it can be used currently by all centres caring for patients with CF. Once statistical tools are established, combined scoring systems with for example clinical items and radiological items can be further developed.

We present in this work the development of statistical tools to create such a scoring system using database information.

## Methods

The diagnosis of CF in individuals in the cohort at the Royal Children's Hospital Melbourne, Australia, is made according to published guidelines [1], with approximately 90% of individuals diagnosed by the new born screening program [8]. The CF cohort at the Royal Children's Hospital represents over 90% of the children in the state of Victoria with a diagnosis of CF [9].

Demographic factors and clinical data as well as pulmonary function and bacterial data of all CF individuals in our cohort are recorded in the Australasian CF registry [8,10]. The main data groupings were categorised in either continuous interval data such as the pulmonary function values, height and weight, number of outpatient visits and days hospitalised, or as binary data such as sputum cultures, complications and antibiotics. Approval for this study was obtained from the Ethics Committee of the Royal Children's Hospital Melbourne, Australia.

### 1. Pre-processing of data and initial approach

All data for the years 1999 – 2003 of all individuals from our cohort were extracted. A data cleaning process then followed, including normalisation of lung function parameters (FEV1 = forced expiratory volume in 1 second, FVC = forced vital capacity and FEF = forced expiratory flow) for sex, age and height, expressed as percent predicted. In addition, body mass index was calculated from weight and height (body mass index = weight in kilograms divided by the square of height in meters) and expressed as percentiles (BMIPCT) using the Centre of Disease Control growth charts for the United States [11].

Initially an unsupervised approach was taken to gain understanding of the groups that may be naturally formed by the data. At this stage the clustering was performed on records rather than individuals. Three clustering algorithms were used: k-means, Kohonen self organising maps [12] and an incremental optimization based clustering algorithm [13]. The incremental algorithm can be applied without pre-specifying a number or nature of clusters. This algorithm produced 5 clusters for the whole data set but tended to produce 4 clusters for males and 3 for females. There was general agreement between the clusters produced using the different algorithms. Decision trees based on C4.5 [14] were used to give rule descriptions of the clusters and these were examined by clinical experts. Although the clusters seemed to make sense there was not a clear way of relating the clusters to a measure of CF severity without going back to knowledge of the individuals. So expert clinical opinion on individuals was obtained and this then allowed a supervised approach to be adopted.

### 2. Data preparation

We decided to utilise, during the learning period of developing the statistical methods, only data of individuals with pulmonary function tests (PFT) in the dataset, as PFT's play an important and well recognized role in the clinical assessment of CF [15]. Performing a PFT does require some compliance and therefore is age dependent, therefore the dataset analysis only included children above the age of 6 years. The last complete entry in the database was considered in the analysis.

Range standardization (each value within a variable is divided by that variable's range) was performed to ensure that variables measured on a larger scale did not unduly dominate the analysis (see below). All binary variables (e.g. 0/1) with > 95% zeros were excluded to achieve a representative dataset with a substantial number of individuals exhibiting the true condition.

The individual's designated paediatric respiratory physician estimated the individual's severity, using a 3-point scale (mild, moderate and severe disease).

A preliminary analysis revealed differences between males and females in terms of how items vary and correlate, consequently separate models were derived to predict male and female CF severity.

### 3. Statistical analysis and technique

Two multivariate techniques were used throughout the analysis to establish the method with better success in feature selection and model derivation: 'Canonical Analysis of Principal Coordinates' (CAP) [16] and 'Linear Discriminant Analysis' (DA) [17]. CAP is a constrained factor analysis method, which attempts to find new axes, based on a linear combination of the original variables, most associated with specified factors or covariates (unlike a unconstrained factor analysis which just tries to explain differences between individuals). CAP, unlike DA, can also account for the ordinality of factors and allows the specification of different measures of inter-individual dissimilarity. In contrast, DA is restricted to measuring factors on a nominal scale and the Manhalanobis dissimilarity coefficient forms an implicit part of this technique. Preliminary analysis revealed that accounting for the ordinal nature of CF severity class (using CAP) lead to higher misclassification rates than if CF severity was considered on a nominal measurement scale (using DA). In the current study, DA is used to determine which variables discriminate between two or more naturally occurring groups.

A 3-step procedure was performed:

(1). Selection of features to identify the variables most associated with changes in the 3-scale severity membership. This was performed by a stepwise procedure of discriminant analysis and also through a process of progressive single feature elimination using optimisation based classifiers. Each feature was eliminated and the effect on classification noted. Only those features that had a substantial effect on classification were retained and the others were identified as candidates for discarding. The list of potentially discardable features agreed with those discarded through the stepwise discriminant analysis process.

(2). Extraction of 5 severity classes out of the 3 expert-opinion severity classes applying Linear DA. As a clinical scale should preferably consist of between 5 and 7 points [18], we have chosen 5 points as it allows to classify patients into groups called "mild", "intermediate moderate", "moderate", "intermediate severe" and "severe".

(3.) Training discriminant functions based on the calibration datasets. This involved two separate Discriminant Analyses: One used to discriminate between mild and moderate cases CF (as classified by clinician); and the other to discriminate between moderate and severe cases. As both of these analyses were two group discriminant analyses, linear discriminant functions were used for classification.

All statistical analyses were conducted using the R (version 2.2) statistics package. Optimization based approaches, developed at the University of Ballarat, Victoria, Australia [19], were also used for feature selection.

### 4. Validation attempt on test dataset

In a final step, we attempted to validate the derived models on a different dataset from individuals from the CF unit at the Royal Children's Hospital Brisbane, Queensland. The data were again extracted from the Australasian CF registry and prepared as described in paragraph 2.

## Results

The sample size of the last complete data entry set included 115 males and 97 females with an age range between 6 and 21 years.

**Figure 1:**
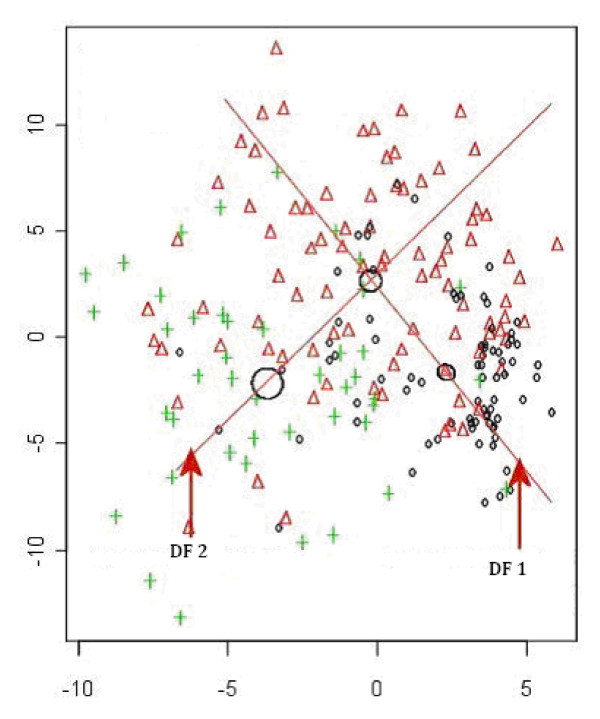
**Linear Discriminant Analysis in contiguous 3 severity groups**. (O = Mild; Δ = Moderate; + = Severe). DA was used to derive the direction in space (a vector), associated with the difference between the groups. DF 1 for mild vs moderate and DF 2 for moderate vs severe. Note: Circles represent regions where each groups mean can be found. The size of the circle indicates each group's variability.

**Figure 2:**
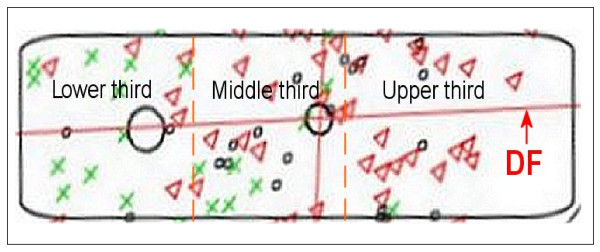
**From 3 to 5 severity groups**. The regions for the three new classes (derived from the original two classes, e.g. mild vs moderate) are chosen such that the number of patients in each group is approximately equal.

**Figure 3:**
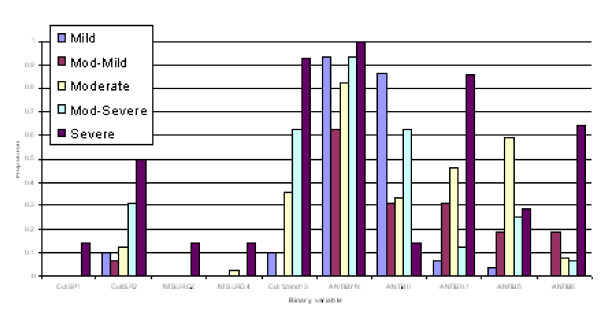
**Profiles of predicted severity groups (Set 1 males)**. The proportion on the Y-axis indicates percent of subjects having that condition in each of the 5 predicted severity groups.

**Figure 4:**
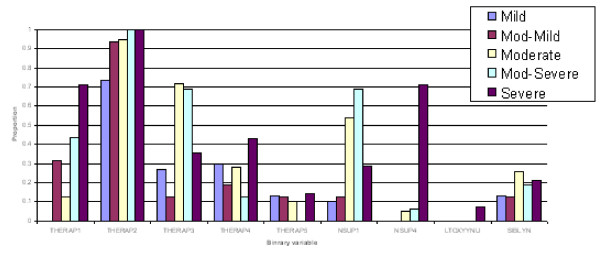
**Profiles of predicted severity groups (Set 2 males)**. The proportion on the Y-axis indicates percent of subjects having that condition in each of the 5 predicted severity groups.

**Figure 5:**
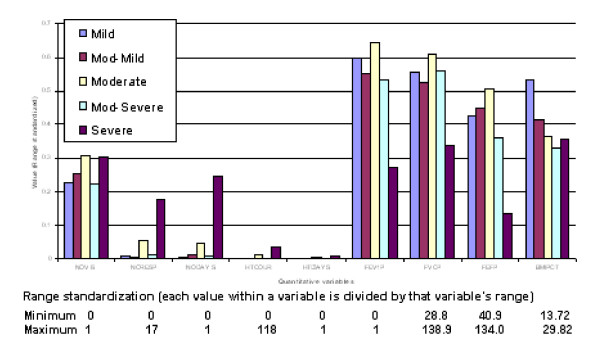
**Profiles of predicted severity groups for quantitative variables (males)**. Mean value for quantitative variables associated with each of the CF severity group.

**Figure 6:**
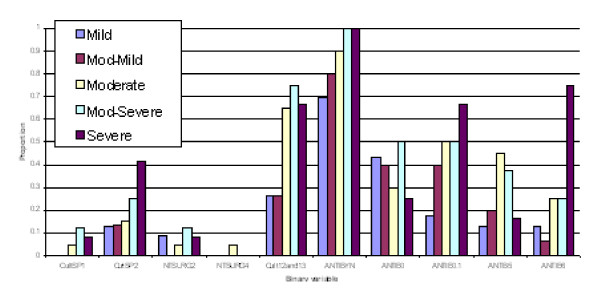
**Profiles of predicted severity groups (Set 1 females)**. The proportion on the Y-axis indicates percent of subjects having that condition in each of the 5 predicted severity groups.

**Figure 7:**
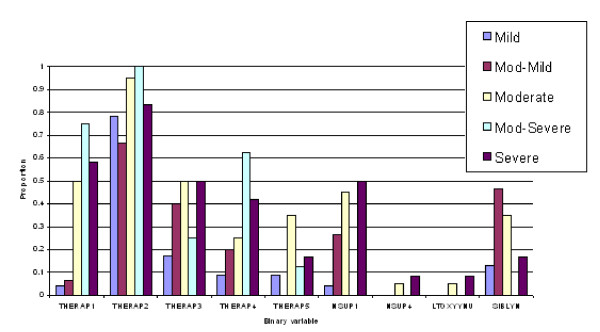
**Profiles of predicted severity groups (Set 2 females)**. The proportion on the Y-axis indicates percent of subjects having that condition in each of the 5 predicted severity groups.

**Figure 8:**
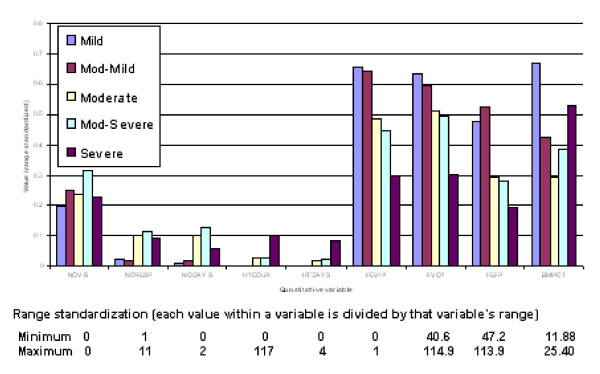
**Profiles of predicted severity groups for quantitative variables (females)**. Mean value for quantitative variables associated with each of the CF severity group.

(1) Feature selection: CAP has a more effective "modelling" focus than DA (data not shown), as it was able to identify the contributing variables (Table 1). Variables not considered by CAP as important in differentiating between CF severities on the 3-point scale were excluded from further analysis. The optimization based approaches resulted in substantial agreement with the variables identified by CAP.

(2) Extraction of 5 severity classes: Variables were identified as important in discriminating contiguous CF severity groups on the 3-point scale as mild/moderate and moderate/severe (Figure 1). For all individuals in the relevant group (e.g. mild and moderate in the first instance); their value was calculated on the appropriate Discriminant Function (DF) to determine whether they occupy the bottom, middle or upper third of the DF (Figure 2). DF1 (the function separating the original mild and moderate groups) was used to derive the three new groups mild, intermediate moderate and moderate; and DF2 (that separating the original moderate and severe groups) to derive the three new groups moderate, intermediate severe and severe. As both analyses contained the moderate group, disagreement between the two discriminant analyses was possible. Hence, posterior probabilities of the individual observations were used to choose which groups the individuals should occupy.

(3) Discriminant functions were trained based on calibration datasets for both males and females and the resulting confusion tables generated (Table 2). As a result, a misclassification rate of 19.1% for males and 16.5% for females was identified, with a majority of misallocations into adjacent severity classes particularly for males. Profiles of predicted severity groups for both males (Figure 3, 4, 5) and females (Figure 6, 7, 8) were drawn. For males, 22 of the 28 selected variables are correlated with the signed severity classes (eg patients with milder disease are less frequent positive for *Pseudomonas aeruginosa *{CULT 12 & 13}. For females, 23 of the 28 selected variables reflect disease severity (eg patients with more severe disease have worse pulmonary function tests.) { FEV1P, FVCP, FEFP}

Validation attempt on test dataset: The misclassification rate was substantially higher with a correct classification in only 34% of males and 23% of females (data not shown).

## Discussion

We present preliminary data for derivation of a scoring system using statistical techniques applied to a CF database. The aim of the present study protocol was to develop statistical methods most likely to be valuable in construction of a scoring system in CF taking into account items regularly and repetitive assessed in all centres caring for patients with cystic fibrosis. The initial unsupervised approach identified a need for an association between classes and CF measure but also suggested that a 5 point scale would be more effective with this data than a finer resolution scale.

A supervised approach was therefore chosen, using CAP and DA in those individuals old enough to perform PFT. The inclusion of PFT parameters in a test dataset was important due to FEV1 being an fairly objective and easily to obtain measurement to assess clinical CF severity and/or improvement in clinical trials. Furthermore, PFT plays a key role in validating a scoring system. BMIPCT as an assessment of therelative proportion of weight for height was introduced, as the percentage of ideal body weight underestimates the severity of malnutrition in CF-children with short stature, whilst overestimating the severity of malnutrition in CF children with tall stature [20].

Throughout the whole analysis, two different statistical methods were used to establish the method with better success: CAP and DA. CAP was used even though it is a multivariate technique very similar to DA as it offers three main advantages:

(1) CAP attempts to detect differences between groups, rather than focusing on how to differentiate between groups (even though mathematically these operations are very similar, the focus of the results is different). (2) DA uses a Manhalanobis measure to differentiate between subjects. In the absence of data preprocessing, variables of a larger magnitude can dominate the analysis (this was another reason we range standardized the data prior to discriminant analysis). In contrast, CAP is not restricted to a particular measure of between-patient differences; therefore it is a much more general technique.

(3) CAP can also account for the ordinal progression of CF severity (i.e. it recognizes the natural order of moving from mild to severe). DA does not recognize the ordinal nature of CF severity class and only considers classes (e.g. severity) on a nominal scale.

The application of CAP was superior to DA in derivation of feature selection; it has a more specific "modeling" focus then DA. In this respect identifying the "contributing variables" is more sensible with CAP than DA. For this to be done with DA, would take a model selection approach to rule out (backwards) or include (forwards) variables. CAP is also more amenable to a graphical representation of the multivariate data. In deriving a model which acknowledged the progressive nature of severity, DA produced lower misclassification rates.

The variables that contribute to predicting severity do not vary with gender. The relative importance (weighting) of the individuals variables however is different between males and females. This may possibly reflect the documented clinical gender differences, where females with CF have been shown across age strata to have a shorter life expectancy [21].

For the calibration dataset, severity classification was accurate for 81% of males and 83% of females, with most of the misallocations into adjacent severity classes. This was encouraging, but unfortunately not repeatable with the test dataset from the Queensland CF unit at the Royal Children's Hospital in Brisbane. One explanation for this constellation might be the small number of individuals (32 in total) in the test dataset, augmented by different treatment strategies and therapeutic approaches between the two centres. Another reason might be that there are actual differences between the calibration (Melbourne) and test (Brisbane) populations, or that measurement and/or treatment protocols vary at the two locations.

A problem in general is that each level or type of therapy is confounded with severity. When individuals get to a certain level of severity, various treatments are prescribed. A canonical analysis model including a temporal (repeated measure) component might help to partition and evaluate the effect of treatment.

There are several limitations of this study. One of the main limitation is the number of data entries, where variables may have been excluded due to too few subjects exhibiting a certain symptom. In addition, the variables identified by CAP may not be the only variables that might be useful for determining CF severity, and that larger samples may identify these additional variables. There also may be other possible explanatory variables that may not have been measured or included in the CF database.

After the initial unsupervised approach failed it was necessary to seek expert clinical opinion on individuals illness severity. An expert opinion is in a way subjective and may not necessarily reflect the real disease severity. Although a clinical scale should preferably consist of between 5 and 7 points [18], to minimize the subjective impact, there was only a classification for the experts into the three groups mild, moderate and severe possible. We then further extracted the preferred 5 groups from the 3 groups (Fig 2) using Linear DA, but this might be one of the reasons the result of the validation study was inconsistent. Linear DA was chosen as both of these analyses, the discrimination between mild and moderate and between moderate and severe cases, were two group discriminant analyses. In addition, using the extracted 5 groups, derived from the original 3 groups of "subjective" expert opinion, as "true" classification for construction of the confusion tables (Table 2), might have problems.

Another main limitation is the lack of validation employing imaging techniques, particularly CT scanning. We have elected not to include radiological aspects into the scoring systems we have explored for several reasons. While some earlier scoring systems did included measurements derived from plain chest X-rays, it is now well established that this form of imaging is an insensitive way of detecting early or mild disease. Nowadays, CT scanning of the lung however has been shown to be significantly more sensitive in detecting early changes of lung disease than plain chest imaging or PFT's [22]. At this stage however CT scanning is not routinely used clinically as an assessment tool for detecting early lung disease in CF as there is a lack of a consensus on the optimal CT scoring system [23], although it is widely used in research studies [24]. In addition, there is a lack of consensus as to what style of CT scanning might be the most appropriate, particularly to reduce radiation dose [24]. Some of the studies have used HRCT (in which thin 0.5–1.5-mm slices are obtained every 0.5, 1, or 2 cm from lung apex to base during inspiration) [25], or volumetric scanning (complete spiral CT imaging covering the entire lung for inspiratory and expiratory scanning) [25] with some recent evidence that only performing exspiratory scanning to reduce radiation exposure might be sufficient to monitor progression of CF disease [26]. Again others are using so called 3 slice scanning protocols to reduce radiation dose with just 3 slices spaced above the carina, below the carina and just above the diaphragm [24]. With this ongoing effort to reduce radiation dose, scanner settings and protocolls will be further altered with still some way to go before CT scans can be used routinely clinically. In addition, there is some emerging evidence that another lung function measurement than PFT's, the lung clearance index by multiple-breath inert gas wash out, may be even more sensitive than HRCT scanning for detecting early lung involvement in CF [27]. This has led us to decide not to include CT scanning in our scoring system. We feel that at this stage it is appropriate to exclude CT scanning as part of any scoring systems. Once fully established, CT scoring may well be a useful addition to future clinical scores, and we agree that this factor then should be included.

## Conclusion

Our preliminary data show that using CAP for detection of selection features and DA to derive the actual model in a CF database is promising for developing a scoring system. More data are needed to finalize a score and the statistical tools have further to be refined and validated in datasets of other centres. For this, we will seek access to additional data from other large cohorts such as the Australasian CF registry. Once a scoring system has been finalized then it will need to be clinically assessed with regard to intra- and inter-observer reliability, and combined scoring systems with for example clinical items and radiological items can be further developed.

## Abbreviations

ANTIBYN: Antibiotic use; ANTIB2: Oral antibiotic use: if YES, was it when required; ANTIB3: Oral antibiotic use: if YES, was it continuous; ANTIB5: Inhalation antibiotic use: if YES, was it when required; ANTIB6: Inhalation antibiotic use: if YES, was it continuous; BMIPCT: Body Mass Index percentiles; CAP: Canonical Analysis of Principal Coordinates; CF: Cystic Fibrosis; CFTR: Cystic fibrosis transmembrane regulator protein; CultSP1: Yeast; CultSP2: Candida albicans; Cult12and13: Pseudomonas aeruginosa, both rough and mucoid; DA: Linear Discriminant Analysis; DF: Discriminant Function; FEV1P: Forced expiratory volume in 1 second as percentage predicted; FVCP: Forced vital capacity as percentage predicted; FEFP: Forced expiratory flow as percentage predicted; HTCOUR: Total number of home-intravenous antibiotic therapy courses; HTDAYS: Total days on home-intravenous antibiotic therapy courses; LTOXYYNU: Long term oxygen therapy; NODAYS: Number of days hospitalised; NORESP: Number of CF respiratory related hospitalisations; NSUP1: Oral nutritional supplements ; NSUP4: Nutritional supplements via Gastrostomy ; NTSURG2: Non-transplant surgery: permanent intravenous access device ; NTSURG4: Non-transplant surgery: Gastrostomy; NOVIS: Number of CF related outpatient clinic visits; PFT: Pulmonary function tests; SIBLYN: Siblings with Cystic Fibrosis; SK-score: Scoring system by Shwachman and Kulczycki; THERAP1: Pulmozyme; THERAP2: ; Pancreatic enzymes; THERAP3: Vitamin Supplements; THERAP4: Bronchodilators; THERAP5: Inhaled cortico-steroids

## Competing interests

The authors declare that they have no competing interests.

## Authors' contributions

GMH participated in the study design and coordination and drafted the manuscript. CH participated in study design, and helped to draft the manuscript. JY participated in the study with his statistical expert background, participated in its design and coordination, and helped to draft the manuscript. JS was involved with database entries, participated in coordination and helped to draft the manuscript. ZD carried out the programming of the data and participated in its design. PJR conceived of the study, participated in coordination and helped to draft the manuscript. RPJ had general supervision of the research group. All authors read and approved the final manuscript.

## Pre-publication history

The pre-publication history for this paper can be accessed here:


